# Prediction of Diabetes through Retinal Images Using Deep Neural Network

**DOI:** 10.1155/2022/7887908

**Published:** 2022-06-03

**Authors:** Mahmoud Ragab, Abdullah S. AL-Malaise AL-Ghamdi, Bahjat Fakieh, Hani Choudhry, Romany F. Mansour, Deepika Koundal

**Affiliations:** ^1^Information Technology Department, Faculty of Computing and Information Technology, King Abdulaziz University, Jeddah 21589, Saudi Arabia; ^2^Centre for Artificial Intelligence in Precision Medicines, King Abdulaziz University, Jeddah 21589, Saudi Arabia; ^3^Mathematics Department, Faculty of Science, Al-Azhar University, Naser City 11884, Cairo, Egypt; ^4^Information Systems Department, Faculty of Computing and Information Technology, King Abdulaziz University, Jeddah 21589, Saudi Arabia; ^5^Information Systems Department, HECI School, Dar Alhekma University, Jeddah, Saudi Arabia; ^6^Center of Excellence in Smart Environment Research, King Abdulaziz University, Jeddah 21589, Saudi Arabia; ^7^Biochemistry Department, Faculty of Science, King Abdulaziz University, Jeddah 21589, Saudi Arabia; ^8^Department of Mathematics, Faculty of Science, New Valley University, El-Kharga 72511, Egypt; ^9^School of Computer Science, University of Petroleum & Energy Studies, Dehradun, India

## Abstract

Microvascular problems of diabetes, such as diabetic retinopathy and macular edema, can be seen in the eye's retina, and the retinal images are being used to screen for and diagnose the illness manually. Using deep learning to automate this time-consuming process might be quite beneficial. In this paper, a deep neural network, i.e., convolutional neural network, has been proposed for predicting diabetes through retinal images. Before applying the deep neural network, the dataset is preprocessed and normalised for classification. Deep neural network is constructed by using 7 layers, 5 kernels, and ReLU activation function, and MaxPooling is implemented to combine important features. Finally, the model is implemented to classify whether the retinal image belongs to a diabetic or nondiabetic class. The parameters used for evaluating the model are accuracy, precision, recall, and F1 score. The implemented model has achieved a training accuracy of more than 95%, which is much better than the other states of the art algorithms.

## 1. Introduction

Diabetes is a condition in which the body's ability to process sugar (glucose) is impaired [1]. Because of this, glucose levels in the blood shoot through the roof. Hyperglycemia is the medical term for this condition [2]. The body is unable to create enough insulin when this occurs. There is also the potential that the body cannot respond to the produced insulin. Diabetes cannot be cured, but it can be managed. Nerve damage, heart attacks, kidney failure, and stroke are possible outcomes for diabetes. Diabetes affects an estimated 8.8% of the world's population, according to statistics from 2017 [3]. By 2045, this figure is expected to rise to 9.9 percent.

Type 1 diabetes (T1D) and type 2 diabetes are the two types of diabetes (T2D) [4]. Most people diagnosed with type 1 diabetes are in their teens or early twenties. High blood glucose levels and increased thirst and urination are the most common symptoms. Oral drugs alone are ineffective in treating this kind of diabetes; hence, insulin therapy is essential. Obesity, hypertension, dyslipidemia, arteriosclerosis, and other disorders are all more common in older adults and the elderly regarding type 2 diabetes [5].

Diabetes is becoming more and more widespread as people's standard of living rises. Diabetes diagnosis and analysis should be studied because of the importance of speed and accuracy. Glucose tolerance, fasting blood glucose levels, and random blood glucose levels are all used to diagnose diabetes in the medical community [6]. The sooner we get a diagnosis, the easier it will be to treat it. Based on a person's daily physical examination data, machine learning can assist humans in making a preliminary diagnosis of diabetes mellitus. The most critical issues in machine learning are identifying useful features and the correct classifier [7].

The standard machine learning methods, such as the support vector machine (SVM), the decision tree (DT), the logistic regression, and others, have recently been applied to predict diabetes [8]. PCA and fuzzy neural inference were used to separate patients with diabetes from those who were not. As a result of the QPSO method and weighted least squares support vector machine (WLS-SVM) developed by Chi et al. [9], type 2 diabetes can be predicted. Diabetes can be predicted using a model developed by Çalişir and Doğantekin, known as LDA-MWSVM [10]. Linear discriminant analysis (LDA) was utilized to reduce dimensionality and extract features in this system [11]. High-dimensional datasets necessitated logistic regression to build prediction models for diverse onsets of type 2 diabetes. SVR (support vector regression) was utilized by Georga et al. to predict diabetes, a multivariate regression problem [12]. To further enhance the accuracy of the results, a growing number of studies are using ensemble approaches [13]. Combining 30 machine learning algorithms, Ozcift and Gulten developed an ensemble approach known as rotation forest [14].

Diabetes can be accurately predicted using AI-based technologies [15]. Deciduous categorization power is one of the advantages of using decision trees in the medical industry. In addition, a random forest produces a large number of decision trees. Recently, neural networks have emerged as a well-known machine learning technique because of their superior overall performance. In this article, deep neural networks will be used to predict the onset of diabetes. The proposed work will use the convolutional neural network to indicate diabetes [16]. In addition, the proposed work has used the dataset consisting of retinal images, and a deep neural network will be implemented on this retinal image dataset to predict the disease [17].

The organisation of the paper is as follows: Section 1 describes the introduction of the paper, whereas Section 2 discusses about the background study. In Section 3, proposed methodology is explained with results and discussion in Section 5. Finally, the conclusion in Section 5 followed by the reference section.

## 2. Related Work

When it comes to saving a person's life, early diabetes diagnosis is critical. In the last several years, a number of new diagnostic methods have been developed based on various models and methodologies. Neural networks, deep learning, and machine learning are just a few of the methods that can be used to improve facial recognition [18]. Other methods include decision making, KNN, retinal pictures, and face images for diagnosis [19].

Joshi and Borse [20] developed a neural network called back propagation (BPNN). MathWorks (MATLAB) was used to create the user-interface. Researchers utilise the Pima Indian Diabetes Dataset to test their proposed methods. Parsing is conducted after the dataset has been loaded. ANNs were trained using back propagation neural networks after reading the values one by one. During the feature extraction phase, values were grouped together based on shared characteristics, and the groups were then arranged in a table. In the proposed method, the following step was to normalise the data. The data were encoded as a binary number between 0 and 1 [21]. Data redundancy is eliminated, and data relationships are ensured, as a result of normalisation. The final phase in the proposed method was training. The proposed system underwent up to nine iterations of training. The third iteration yielded the lowest level of error. At lower epoch values, the best results were achieved. Regression and validation plots were used to generate the results but accuracy is not.

The computational speed and efficiency of feed forward ANN (FFANN) make it popular in today's society. Diagnosis of diabetes can be improved by using the Small World FANN model, according Erkaymaz and Ozer [22]. Researchers considered a four-layered FFANN in their investigation. The network included one output neuron and eight inputs. In FFANN, they made use of two hidden layers. FFANN used two alternative network topologies. Scientists rely on a bipolar-sigmoid function as their activation function in developing the new approach. The SW-FFANN training algorithm was based on a backpropagation learning algorithm. The PIDD dataset from the University of California, Irvine (UCI) repository was used in this study. The drawback is that optimum regular topology for SW-network development had been used in the rewiring procedure, for which DGlobal and DLocal parameters were determined.

A technique based on artificial neural networks was described in detail. Input, hidden, and output layers make up the three main components of an artificial neural network. Raw data are sent to the input layer. Inputs and weights assigned to them determine how hidden layers work. The data were entered into a JNN tool that calculates the attributes' values. Training, testing, and validation of data were then carried out–Binary numbers were the output of the suggested system. As a diabetic, I scores 0 points, while a healthy one scores 1. The proposed system had an average error rate of 0.010. The dataset underwent a total of 158,000 iterations. There were 767 training samples and 237 validation samples. The limitation of this technique is that its computationally complex.

Aliberti et al. [23] examined the prediction algorithms trained on glucose signals from a large and heterogeneous cohort of patients and then applied them to estimate future glucose levels on a brand-new patient. Based on nonlinear autoregressive (NAR) and long short-term memory (LSTM) neural networks, the authors have developed and compared two different types of solutions that have been successful in numerous time series prediction situations [24].

A deep neural network framework based on stacked autoencoders was presented by Kannadasan et al. [25] to classify the diabetes data. First, stacked autoencoders are used to extract features from the dataset, and the dataset is then categorised using a softmax layer. Finally, the network is fine tuned using the training dataset using supervised backpropagation. Pradhan et al. [26] used skin impedance and heart rate variability to identify diabetes. Classification was accomplished with the usage of artificial neural networks. Six females and five males with diabetes, an average age of 8 to 40 years, had been studied for skin impedance data. In addition, data from eight normal people, five females, and three males, with an average age of 3 to 24 years, were gathered for the study. When it came to determining signal strength at various frequencies, the Welch method was employed. Data on the electrocardiograms of 20 healthy volunteers, 14 men, and 6 women, with an average age of 22 years and 7 months, were gathered. Additionally, information was gathered on 20 diabetes patients, eight of whom were female and twelve of whom were male, with an average age of 40 years and eight months. In order to remove baseline drift from the resulting signal, median filtering was employed during the initial stages of signal preprocessing. Butter worth a lowpass filter was also used to reduce the high-frequency noise. The Savitzky–Golay filter was then used to smooth the ECG signal [27].

A deep neural network screening model was created by Ryu et al. [28] for patients with undetected diabetes mellitus (DM). Data from the Korean National Health and Nutrition Examination b (KNHANES) from 2013–2016 were used in our cross-sectional investigation. Only 11,456 people were included in the study after removing those diagnosed with DM, those under the age of 20, and those with incomplete data. KNHANES 2013–2015 was utilised as a training dataset and evaluated to generate a DLM for undiagnosed diabetes mellitus. The DLM was tested on a sample of 4,444 people who completed the 2016 KNHANES survey. Age, waist circumference, BMI, gender, smoking status, hypertension, and family history of diabetes (FH) were used to build the DLM. The area under the curve (AUC) of the model was 80.11, which is in line with previous screening models' performance [29].

## 3. Materials and Methods

**Figure 1:**
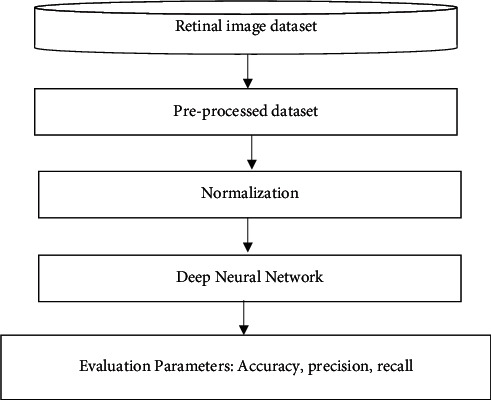
Flowchart of the proposed work.

The proposed methodology has been discussed in this section. Multilayer neural networks have been employed in the suggested research as a deep NN [30]. Convolutional neural networks are gaining popularity as data are structured as an image. Normalization is a key part of this procedure, as it is used for most of the data. Before beginning any work, it is highly advised to preprocess the images from dataset. As a result, preprocessed data will help in improved accuracy. This dataset has been fed into proposed deep convolutional neural network after preprocessing and normalization. Deep neural networks (DNNs) are then used to run and fit our data, resulting in the output. The following sections will provide a high-level overview of the completed work.  Figure 1 displays the flowchart of the proposed model.

### 3.1. Dataset

The images used for performing the analysis is downloaded from the Github https://github.com/deepdrdoc/Deep-Diabetic-Retinopathy-Image-Dataset-DeepDRiD. In this work, 410 images based on retina are used for performing the analysis and these images are classified for predicting the images as diabetic or nondiabetic.

### 3.2. Preprocessing Data

As previously stated, preprocessing is critical to this work. Image processing techniques are used to perform preprocessing on this dataset. To do this, the approaches described above have been employed to locate and bold the intensity of aberrant locations and parts. As a result, an unusual structure can be seen in some images. The optic disc and vessels, as an example, are not typical. The findings of previous tests using multichannel images were not very dependable; this was the case even before attempting a solution based on grayscaled data. As a result, grayscale graphics were chosen as the preferred method of presentation. The next step is to normalize the photos after converting them to grayscale.

In the preprocessing stage, data may now be normalised easily by dividing image intensities to 255 (image converted to greyscale previously). The data must first be normalised using a label to form the network. Each picture name contains a class label in the first substring. The preprocessed data have been standardised as of this moment.

### 3.3. Creating Deep Neural Network Model

This section discusses deep neural networks with seven layers using different activation functions. For example, the first layer of the convolutional1D network uses the ReLU activation function with a kernel size of 5. After preprocessing, the data are reduced to a 256*∗*256 grayscale picture [31]. The input shape of size (256, 256, 1) will be utilized as an input. After that, MaxPooling combines the most important features, then flattens the image, and finally, the classification will be done. The dense layer should be utilized because we have binary categories (diabetic vs. nondiabetic). Because our class labels are binary, our loss function is binary cross-entropy. Finally, Adam's batch size is set to 10, and it is used as an optimizer in batch mode. This optimizer will help to prevent the overfitting of the proposed model [32].

**Figure 2:**
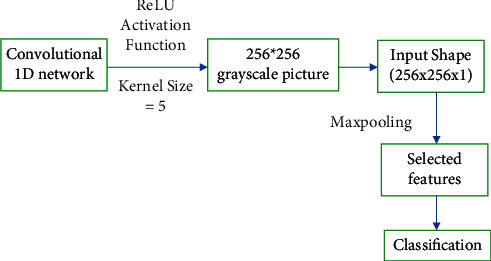
Architecture of deep neural network.

**Figure 3:**
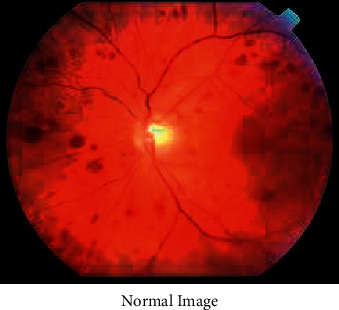
Normal image.

Figure 2 has shown the architecture of the proposed neural network with the size representation of the images. The Figure 3 has also shown the use of activation function and the max pooling layer for performing the classification.

## 4. Results and Discussion

**Figure 4:**
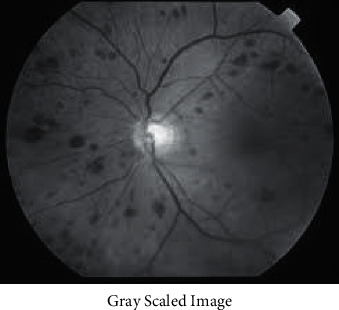
Gray scaled image.

**Figure 5:**
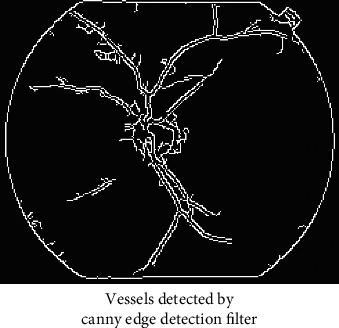
Vessels detected by canny edge detection filter.

**Figure 6:**
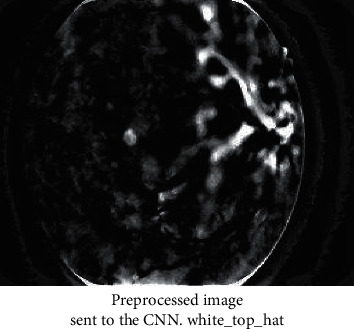
Preprocessed image sent to the CNN. white_top_hat + gray_scaled.

The proposed work is implemented using the anaconda framework for executing the Python codes. Anaconda framework is rich in libraries based on deep learning due to which TensorFlow and Keras were easily imported in the code. Different aspects of the image taken for the analysis are given below:

Figures 3–6 has shown the different shades of the images. Figure 3 has represented the normal image, whereas Figure 4 has illustrated the gray scaled image.  Figure 5 has shown the vessels detected by canny edge detection filter, and at last, Figure 6 has shown the generated preprocessed image which is further classified by the deep neural network.

Above Tables 1 and 2 has shown the comparison chart and confusion matrix of the proposed work, respectively. In Table 1, the proposed deep neural network is compared with the existing machine learning algorithms and the results have indicated that the proposed work has performed better than the existing machine learning algorithms.

## 5. Conclusion

Diagnosing diabetes at an early stage is critical to finding an effective treatment. Diabetes classification is implemented using a deep neural network, i.e., convolutional neural network, in the current work. The dataset contains more than 410 images based on the retina for diabetes classification. The number of training epochs was kept short of ensuring that the approach could be quickly used on any mobile device. The experimental results suggest that the proposed deep learning model is effective and accurate. The model has achieved an accuracy greater than 95%. The model for determining all probable complications, including an orderly sequence in terms of the percentage of complications that can occur, will be improved in a future study as well. Additionally, deep learning algorithms and methodologies can be incorporated to enhance the work for an automated diabetes analysis.

## Figures and Tables

**Table 1:** Comparison chart of the proposed work.

Model	Class-label	Precision	Recall	*F*1-score	Accuracy (%)
Logistic regression [33]	0 (nondiabetic)	0.72	0.71	0.93	73
	1 (diabetic)	0.73	0.72	0.94	

Random forest	0 (nondiabetic)	0.76	0.75	0.75	77.4
	1 (diabetic)	0.75	0.77	0.76	

Proposed fine-tuned MLP	0 (nondiabetic)	0.86	0.85	0.85	86.6
	1 (diabetic)	0.86	0.88	0.87	

Deep neural network (proposed)	0 (nondiabetic)	0.95	0.91	0.93	95.6
	1 (diabetic)	0.94	0.93	0.94	

**Table 2:** Confusion matrix.

	True	Positive
False	0.95	0.91
True	0.94	0.93

## Data Availability

Data are publicly available at https://github.com/deepdrdoc/Deep-Diabetic-Retinopathy-Image-Dataset-DeepDRiD.
